# A cross-sectional study of essential surgical, obstetric, and anaesthesia care capacity in the public sector in Fiji

**DOI:** 10.1371/journal.pgph.0003829

**Published:** 2025-02-05

**Authors:** Ashneel Sundar, Jope Makutu, Ifereimi Waqainabete, Grace Zhang, Jemesa Tudravu, Josese Turagava, Kiki Maoate, Rajeev Patel, Rennie Xinrui Qin

**Affiliations:** 1 Department of Surgery, Colonial War Memorial Hospital, Suva, Fiji; 2 Umanand Prasad School of Medicine and Health Sciences, The University of Fiji, Suva, Fiji; 3 Kellogg Institute for International Studies, University of Notre Dame, Notre Dame, Indiana, United States of America; 4 Ministry of Health and Medical Services of Fiji, Suva, Fiji; 5 Department of Paediatric Surgery, Christchurch Hospital, University of Otago, Christchurch, New Zealand; 6 The Program in Global Surgery and Social Change, Department of Global Health and Social Medicine, Harvard Medical School, Boston, Massachusetts, United States of America; 7 Department of Epidemiology and Biostatistics, Faculty of Medical and Health Sciences, University of Auckland, Auckland, New Zealand; University of Global Health Equity, RWANDA

## Abstract

The Lancet Commission on Global Surgery indicator collection highlighted gaps in surgical, obstetric, and anaesthesia (SOA) care in Fiji. Our study is the first comprehensive assessment of essential SOA care capacity in Fiji to guide national surgical planning. In February 2021, we conducted a cross-sectional survey of public hospitals in Fiji using the World Health Organization-Program in Global Surgery and Social Change surgical assessment tool. We surveyed 18 facilities, including three divisional hospitals (DHs) and 15 subdivisional hospitals (SDHs). Twenty-two functional operating rooms (ORs) and 27 post-anaesthesia care beds served 884,887 people. Surgical care was concentrated in DHs and only delivered in select SDHs during outreaches. While SDHs had OR space, they lacked equipment, supplies, and human resources. From 2016 to 2021, surgical volume per 100,000 population increased by approximately 50% from 1,490 to 2,248; however, SOA specialists density per 100,000 population increased marginally from 5.8 to 7.1. There is significant variation across divisions. The Northern division has more ORs (4.55), SOA specialists (9.1), and surgical volume (3,731) per 100,000 population than the Central (2.40, 7.9, 2,367) and Western (1.78, 5.3, 1,519) divisions. This is due to more OR space and functioning, specialist post creation, and outreach services to SDHs. Policy recommendations include upgrading key SDHs with essential SOA care capacity, growing the SOA workforce, strengthening facility maintenance and climate resilience, and strengthening outreach programs. Investment in surgical care capacity must be urgently increased to meet the population’s needs.

## Introduction

The World Health Assembly resolution 68.15 recognised emergency and essential surgical and anaesthesia care as indispensable to universal health coverage [1]. The Pacific Health Ministers echoed this recognition and championed the development and implementation of National Surgical, Obstetric, and Anaesthesia Plans (NSOAPs) towards achieving the Healthy Island Vision [2].

The Pacific region comprises 22 countries and territories surrounded by 165 million km^2^ of ocean [3]. Pacific Island Countries (PICs) face unique geographic barriers to surgical care, including their small populations, geographic dispersion, distance from the global economy, and vulnerability to climate change [3,4]. This has resulted in challenges with supply chains, the maintenance of equipment and human resources, and the provision of specialised care [5,6]. The Lancet Commission on Global Surgery (LCoGS) indicators collection in 13 PICs in 2016 demonstrated challenges in surgical care access, specialist workforce, surgical volume, and financial risk protection [7].

Fiji is the largest nation in the South Pacific and an upper-middle-income country with a population of 887,884 and a GDP per capita of $5,086 US dollars [8]. Since early history, Fiji has served as a regional hub for cultural exchange, trade, and transportation. The British Empire colonised Fiji from 1874 to 1970 and expanded the sugar industry with indentured labourers [9]. Its population has grown from 527,000 in 1970 and consists of 62% Indigenous Fijians, 31% Indo-Fijians, and 7% other groups [8,10].

Similar to other PICs, Fiji faces a high burden of diseases requiring surgical, obstetric, and anaesthesia (SOA) care, including traumatic injuries, infectious diseases (rheumatic heart disease and human papillomavirus), non-communicable diseases (diabetic foot sepsis and retinopathy), and maternal mortality [11–15]. Surgical care capacity assessments have been conducted in Vanuatu, Samoa, Solomon Islands, and Papua New Guinea [4,16–18]. However, little is known about the SOA care capacity in Fiji.

Most previous surgical facility assessments have been conducted by researchers from the Global North [19,20]. Global health studies conducted from a Global North gaze have been found to carry epistemic biases, which challenges internal validity [21].

In this study, we aim to assess the national SOA care capacity in public facilities in Fiji from a local provider’s perspective to guide policy development towards surgical system strengthening.

## Materials and methods

### Study design

In 2021, the Fiji Ministry of Health and Medical Services (MoH) began developing an NSOAP. As part of this policy initiative, the MoH conducted a mixed-method cross-sectional assessment of SOA care capacity in Fiji. From December 2022 to December 2023, we accessed the quantitative component of this policy data for secondary research purposes.

### Study setting

**Fig 1:**
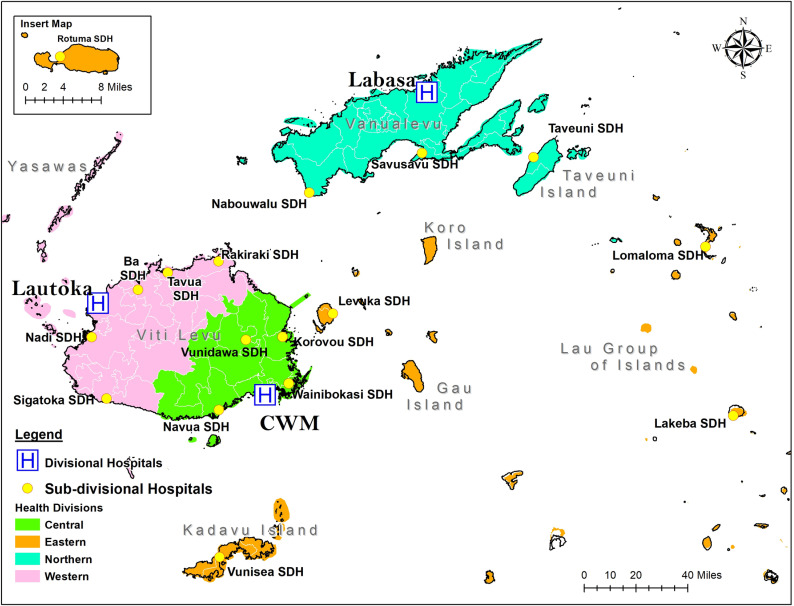
Divisional and subdivisional hospitals in Fiji, 2021. The base map used to construct the map image was obtained from ArcGIS (Esri): https://www.arcgis.com/apps/mapviewer/index.html?webmap=59bd205db62243759cf3e47a8a6ad639.

Fiji’s health system is predominantly tax-funded and publicly delivered [22]. The MoH stratifies public facilities by geographic division (Central and Eastern, Northern, and Western) and level of care (divisional, subdivisional, health centre, and nursing station) [23]. Three divisional hospitals (DHs) provide specialised clinical care. Seventeen subdivisional hospitals (SDHs) provide 24-hour generalised clinical services and supervise health centres and nursing stations in designated medical areas. Fig 1 contains a map of health facilities. Different levels of care are connected through clinical services outreach [23]. SOA specialists travel from DHs to deliver outpatient consultations and elective surgeries in SDHs.

Ninety-three percent of its population lives on the two main islands of Viti Levu and Vanua Levu, and the remaining in the maritime province of the Eastern division [8]. Colonial War Memorial (CWM) Hospital, the national referral hospital, serves the Central and Eastern division, and Lautoka Hospital serves the Western division. They are situated on Viti Levu, with 81% of Fiji’s population [24]. Labasa Hospital serves the Northern division and is situated on the second largest island, Vanua Levu, with 15% of Fiji’s population. DHs served a population of between 100,000 and 400,000. SDHs served a population of between 1,500 to 76,000. SDHs in the Northern and Eastern divisions served a smaller population of 1,500 to 34,000 compared to 18,000 to 76,000 in the Central and Western divisions, highlighting their geographic dispersion and remoteness.

The private sector is rapidly expanding, with four private hospitals provided limited elective SOA care in urban areas. Private expenditures comprised 22.0% of hospital expenses in 2021 [25].

### Participants

We included all DHs and SDHs in the public system and excluded health centres and nursing stations.

### Data collection

Data were collected using the validated World Health Organization- Program in Global Surgery and Social Change surgical assessment tool (WHO-PGSSC SAT, S1 Appendix). It contains 169 questions based on the WHO Health Systems Building Blocks Framework: infrastructure, workforce, service delivery, information management, and finance [26].

The MoH contacted each facility. Senior medical providers administered the tool in each facility after undergoing training. Data was collected in February 2021 through hospital walk-throughs, review of operative logbooks, admission and outpatient records, direct observation of consumable and medication availability, and staff interviews. Only anonymised data and no individually identifiable data were collected.

The availability of utilities (electricity, running water, internet, oxygen), equipment, consumables, and medication was measured using a 6-point scale: 100%, 76–99%, 51–75%, 26–50%, 1–25%, and 0% of the time. Surgical volume was collected over a 12-month period preceding the data collection date.

### Definitions

Bellwether procedures serve as an indicator of essential surgical care capacity and include caesarean section, laparotomy, and surgical management of open fractures [27]. Surgical procedures were categorised as primary procedures delivered at primary health centres, secondary procedures delivered at first-level hospitals, and tertiary procedures delivered at referral hospitals according to the Disease Control Priorities third edition (DCP3) and previous literature (S1 Table) [28,29]. Timely blood transfusion was defined as access to blood transfusions within two hours.

### Data analysis

Stratifying facilities by level and division, descriptive summary statistics were calculated on the number of hospital beds, operating rooms (ORs), post-anaesthesia care unit (PACU) beds, intensive care unit (ICU) beds, anaesthetic machines, pulse oximeter, specialist and non-specialist SOA providers, and surgical volume. Population density of ORs, PACUs, ICU beds, SOA specialists, and surgical volume were calculated by division. Heat maps were used to display the availability of utility, blood, diagnostics, medication, equipment, and supplies. The in-hospital peri-operative mortality rate (POMR) was calculated by dividing the number of in-hospital deaths by the total surgical volume over 12 months. The caesarean section rate was calculated by dividing the number of caesarean sections by the total number of deliveries over 12 months. We generated an update for three of the six LCoGS indicators at a national level, including surgical volume, SOA specialists, and POMR [27]. Missing or unclear data fields were validated with submitters by email or phone. The results were triangulated with the qualitative data during stakeholder meetings as a part of the NSOAP process.

### Ethics

Ethics approval was granted by the Fiji Human Health Research Ethics Review Committee (FHHRERC) under the MoH and the Harvard Longwood Campus Institutional Review Board (IRB21-122). Formal verbal consent was obtained from the Medical Officer at each facility to conduct the survey.

## Results

### General

Eighteen out of 20 eligible public facilities were included, encompassing three DHs and 15 SDHs. Two SDHs, Tavua and Wainibokasi, were excluded due to non-response after multiple contact.

### Infrastructure

#### Beds and space.

Table 1 provides an overview of the SOA care infrastructure in Fiji. Three DHs and 15 SDHs with 1,444 beds, 22 functional ORs, 27 PACUs, 30 ICU beds, 39 functional ventilators, and 28 anaesthetic machines served a population of 884,887. Nationally, 2.48 functional ORs, 3.05 PACU beds, and 3.39 ICU beds were available per 100,000 population.

**Table 1:** The availability of surgical, obstetric, and anaesthesia infrastructure in surveyed health facilities, Fiji, 2021.

Facility level	Name	Catchment population	Beds	Functional ORs^*^	OR per 100,000 population	PACU beds	PACU per 100,000 population	ICU beds	ICU per 100,000 popu-lation	Venti-lators	Anaesthetic machines	Pulse oximeter
Central & Eastern division		415,932	599	10	2.40	16	3.85	16	3.85	25	12	48
DH	CWM	415,932	458	5		14		16		25	9	20
SDH	Navua	27,895	20	1		0		0		0	0	5
	Korovou	22,649	12	1		1		0		0	0	5
	Vunidawa	17,769	22	1		0		0		0	1	5
Eastern division												
	Levuka	15,657	32	1		1		0		0	1	3
	Vunisea	10,869	22	1		0		0		0	0	1
	Lakeba	4,642	12	0		0		0		0	1	5
	Lomaloma	2,781	16	0		0		0		0	0	1
	Rotuma	1,583	5	0		0		0		0	0	3
Western division		337,041	560	6	1.78	5	1.48	11	3.26	11	8	17
DH	Lautoka	337,041	339	3		3		11		11	4	10
SDH	Nadi	75,838	86	1		0		0		0	2	0
	Ba	72,582	50	0		0		0		0	0	5
	Sigatoka	58,940	56	1		2		0		0	1	2
	Rakiraki	30,416	29	1		0		0		0	1	0
Northern Division		131,914	285	6	4.55	6	4.55	3	2.27	3	8	31
DH	Labasa	131,914	170	3		4		3		3	5	20
SDH	Savusavu	33,660	56	1		0		0		0	1	5
	Taveuni	16,787	33	1		0		0		0	1	1
	Nabouwalu	15,489	26	1		2		0		0	1	5
All DHs			967	11		21		30		39	18	50
All SDHs			477	11		6		0		0	10	46
National 884,887			1444	22	2.48	27	3.05	30	3.39	39	28	96

* CWM Hospital had eight total ORs, five of which were functional. Lautoka Hospital had six ORs, three of which were functional.

CWM, Colonial War Memorial; DH, divisional hospital; ICU, intensive care unit; OR, operating room; PACU, post-anaesthesia recovery unit; SDH, subdivisional hospital.

Hospital and PACU beds were concentrated in DHs compared to SDHs. ICU beds and ventilators were limited to the DHs. Functional ORs, anaesthetic machines, and pulse oximeters were more evenly distributed between DHs and SDHs. Eleven (73%) SDHs had functional ORs; nine (60%) had anaesthetic machines.

There was a greater than two-fold variation in the population density of OR, PACU, and ICU beds across the divisions. The numbers of functional ORs and PACUs per 100,000 population were the highest in the Northern division, followed by the Central and Eastern division and the Western division (Table 1). Only eight (57.1%) ORs at CWM and Lautoka hospitals were functional. The population density of ICU beds was the highest in the Central and Eastern division, where the national referral hospital is located, followed by the Western division and the Northern division.

#### Utility and diagnostics.

All DHs had consistent access to electricity, running water, oxygen, internet, and basic laboratory studies (Table 2). Thirteen (86.7%) SDHs had an uninterrupted supply of electricity, running water, oxygen, and internet.

**Table 2:** The availability of utilities, blood transfusion, and diagnostics in surveyed health facilities, Fiji, 2021.

Facility	Population	Utilities				Blood transfusion	Radiology				Laboratory				
		Electricity	Running water	Oxygen	Internet		Xray	USS	CT	MRI	CBC	UEC	Coagulation	Urine	Infectious screen
**Divisional hospitals (DHs)**															
CWM	415,932														
Lautoka	337,041														
Labasa	131,914														
**All DHs**		3/3	3/3	3/3	3/3	2/3	3/3	3/3	1/3	1/3	3/3	3/3	3/3	3/3	3/3
**Subdivisional hospitals (SDHs)**															
**Central division**															
Navua	27,895														
Korovou	22,649														
Vunidawa	17,769														
		100%	100%	100%	100%	0%	33%	100%	0%	0%	33%	33%	0%	0%	33%
**Eastern division**															
Levuka	15,657														
Vunisea	10,869														
Lakeba	4,642														
Lomaloma	2,781														
Rotuma	1,583														
		60%	80%	100%	60%	0%	0%	60%	0%	0%	40%	0%	0%	0%	40%
**Western division**															
Nadi	75,838														
Ba	72,582														
Sigatoka	58,940														
Rakiraki	30,416														
		100%	100%	100%	100%	100%	50%	100%	0%	0%	75%	25%	75%	75%	50%
**Northern division**															
Savusavu	33,660														
Taveuni	16,787														
Nabouwalu	15,489														
		100%	100%	100%	67%	0%	100%	100%	0%	0%	100%	33%	100%	67%	67%
**All SDHs**		13/15	14/15	15/15	12/15	4/15	6/15	13/15	0/15	0/15	9/15	3/15	6/15	5/15	7/15

Red, not available (0%); Yellow, occasionally available (1–75%); Green, always available (76–100%).

CBC, complete blood count; CT, computerised tomography; CWM, Colonial War Memorial; MRI, magnetic resonance imaging; UEC, urea and electrolytes; USS, ultrasound.

The availability of diagnostics was variable. Computerised tomography (CT) scans and magnetic resonance imaging (MRI) were only available in one (33.3%) DH, the national referral hospital, and none of the SDHs. The other two DHs had to access CT and MRI through the national referral and private hospitals. Six (40%) SDHs had consistent access to xray, and 13 (86.7%) had consistent access to ultrasound. Most basic laboratory tests were consistently available in less than 50% of SDHs.

Access to timely blood transfusions was poor. Only two (66.7%) DHs and four (26.7%) SDHs, all in the Western division, could consistently access blood transfusion within two hours.

Comparing the divisions, SDHs in the Eastern division had more interruptions in electricity, water, and internet supply. Radiology and laboratory tests were less available in SDHs in the Central and Eastern divisions compared to the Northern and Western divisions.

#### Medication and supplies.

All DHs had consistent access to SOA equipment, supplies, and medications. However, they were of limited availability in SDHs. Among 15 SDHs, antibiotics were consistently available in 12 (80%), sterile gloves in 10 (66.7%), sutures in eight (53.3%), inhalational anaesthesia in three (20%), and chest drains in two (13.3%) (S2 Table).

### Workforce

Table 3 illustrates the SOA workforce. There were 63 full-time equivalent (FTE) SOA specialists across public hospitals, including 25 surgeons, 21 obstetricians and gynaecologists, and 17 anaesthetists. This yielded a national SOA specialist density of 7.1 per 100,000 population in the public sector.

**Table 3:** Total number and density of surgical, obstetric, and anaesthesia care workforce in surveyed health facilities, Fiji, 2021.

		Central & Eastern division		Western division		Northern division	
		DH	SDH	DH	SDH	DH	SDH
Surgeons		17	0	4	0	4	0
Obstetricians and gynaecologists		9	0	8	0	4	0
Anaesthetists		7	0	6	0	4	0
Trainees in Surgery		19	0	11	0	9	0
Trainees in Obstetrics		14	0	13	0	10	0
Trainees in Anaesthesia		13	0	11	0	5	0
Midwives		36	44	39	36	23	29
Pharmacists		13	4	4	5	16	1
Biomedical technicians		6	2	4	0	5	0
Radiologist		1	0	1	0	0	0
Pathologist		1	0	1	0	0	0
By division	Total SOA	33		18		12	
	SOA density^*^	7.9		5.3		9.1	
Total	Total SOA	63					
	SOA density^*^	7.1					

DH, divisional hospital; SDH, subdivisional hospital; SOA, surgeon, obstetrician and gynaecologists, anaesthetists.

* SOA density = the number of SOA specialists per 100,000 population.

There has been development of a subspecialty surgery workforce. Consultant surgeons were available in several subspecialties in the national referral hospital (S3 Table). There were 40 trainees in surgery, 37 in obstetrics and gynaecology, and 29 in anaesthesia.

Workforces with specialised skills, such as SOA specialists, trainees, surgical nurses, biomedical technicians, radiologists, and pathologists, were concentrated in the DHs. Two pathologists and two radiologists served the country.

Comparing the divisions, the SOA population density was the highest in the Northern division (9.1), followed by the Central and Eastern division (7.9) and the Western division (5.3).

### Service delivery

The national surgical volume per 100,000 population is 2,247 (Table 4). The vast majority of surgeries were performed in DHs. All DHs were Bellwether capable, whereas only two (13.3%) SDHs provided Bellwether procedures during specialist outreach during the study period.

**Table 4:** Total surgical volume, case mix, and service quality and safety among surveyed health facilities, Fiji, 2021.

Facility	Population	Surgical volume					Case mix			Quality & safety	
		Laparotomy	Caesarean section	Open fracture repair	Total surgery	Surgical volume per 100,000 population	Primary	Secondary	Tertiary	Caesarean section rate	POMR
Central and Eastern division	415,932	480	1,868	144	9,845	2,367	80.4%	18.8%	0.9%	15.4%	0.2%
DH		480	1,868	144	9,800		71.9%	26.9%	1.3%	18.1%	0.2%
SDH		0	0	0	45		96.8%	3.2%	0.0%	0.0%	0.0%
Western division	337,041	230	1,150	132	5,120	1,519	87.8%	12.1%	0.0%	13.2%	0.2%
DH		228	1,128	132	5,028		73.6%	26.3%	0.1%	18.7%	0.2%
SDH		2	22	0	92		99.0%	1.1%	0.0%	0.8%	0.0%
Northern division	131,914	120	421	720	4,922	3,731	76.7%	23.2%	0.1%	8.6%	0.5%
DH		120	420	720	4,800		73.1%	26.7%	0.1%	14.7%	0.5%
SDH		0	1	0	122		84.6%	15.4%	0.0%	0.0%	0.0%
National	884,887	830	3,439	996	19,887	2,247	82.6%	17.0%	0.4%	13.2%	0.3%

DH, divisional hospital; POMR, perioperative mortality rate; SDH, subdivisional hospital.

In terms of case mix, SDHs were limited to providing primary care procedures. Secondary procedures were mostly delivered in DHs, except during outreaches in the Northern division. Tertiary procedures accounted for 0.4% of the total surgical volume and were almost exclusively delivered at the national referral hospital in the Central division.

POMR is less than 1% across all divisions. The caesarean section rate was 13.2% nationally. It was the highest in the Central and Eastern division (15.4%), followed by the Western division (13.2%) and the Northern division (8.5%).

Comparing the divisions, the Northern division had the highest surgical volume density per 100,000 population (3,731), followed by the Central and Eastern division (2,367) and the Western division (1,519). SDHs in the Northern division had a higher surgical volume density and delivered a greater proportion of secondary procedures than other divisions.

S4 Table lists the ten most commonly performed procedures in Fiji. Amputations, commonly performed for diabetic foot sepsis, ranked among the top four, alongside the three Bellwether procedures: laparotomy, open fracture management, and caesarean section.

## Discussion

This study is the first comprehensive SOA care capacity assessment in public facilities in Fiji. We identified significant gaps in infrastructure, workforce, and service delivery. Compared to the previous LCoGS indicator collection in 2016, surgical volume per 100,000 population has increased by more than 50% from 1,490 to 2,248. SOA specialist density per 100,000 population has only increased marginally from 5.8 to 7.1 [7]. Both indicators were well below their targets of 5,000 and 20, respectively [27]. Variations in surgical capacity by geographic division and care level reveal both the reasons behind the gaps and potential solutions.

The Northern division had the greatest population density of ORs, SOA specialists, and surgical volume compared to the Central and Eastern, and Western divisions. This is due to more specialist post creation and regular outreach in the Northern division and poor OR maintenance and functioning in the Central and Western divisions. Only eight out of 14 ORs in the central and Western divisions were functional due to air conditioning failure and fire.

SOA care delivery was predominantly concentrated in the DHs rather than SDHs. DHs lacked functional ORs, while SDHs had ORs but lacked human resources, diagnostics, and consumables. SDH capacity varied by geographic division. SDHs in the Eastern division experienced more interruptions in utility and diagnostics due to their remoteness and reliance on generators and solar power. SDHs in the Central division had lower diagnostics availability due to over-reliance on the national referral hospital.

Similar to other PICs, Fiji experienced gaps in the provision of specialised services, including radiology, pathology, blood bank, ICU, and subspecialty surgery [3]. Deficits in diagnostics, blood services, and consumables pointed to the common issue of supply chain. For example, ultrasound was more commonly available than xray in SDHs due to its lower reliance on consumables and films. However, Fiji had a greater capacity for specialised services than other PICs [4,5,17]. Growth in a subspecialty surgery workforce in recent years underscores Fiji’s potential as a regional referral centre.

In terms of the quality of surgical care, POMR was less than 1% across all divisions; however, it was not prospectively monitored. Caesarean section rate is within the WHO recommended range of 10-15% nationally [30]. It is above this range in the Central and Eastern division and below this range in the Northern division, likely reflecting the centralisation of high-risk pregnancies.

Fiji shares challenges similar to those of other PICs, including geographic dispersion, growing population and disease burden, and climate change [4,5,17]. The increase in surgical volume from 2016 to 2021 was not matched by a similar proportional increase in SOA specialists. Surgical care capacity assessments in Vanuatu and Samoa also identified increasing constraints on existing infrastructure and workforce due to population growth and service demand [4,5,17]. Amputation, commonly due to diabetes, was the third most common procedure in Fiji, consistent with the substantial NCD burden in other Pacific studies [31,32]. Strengthening SOA workforce capacity, first-level hospital capacity, outreach programs, and regional collaboration have been proposed as solutions by other Pacific studies. [4,5,17]. Although Fiji is an upper-middle-income country, its current health expenditure (CHE) is 3.8% of its GDP, and its life expectancy is 68 years, lower than some of its less affluent Pacific neighbours [3,33,34]. This highlights the importance of adequate investment in healthcare in proportion to population needs.

### Recommendations

Fiji’s surgical care capacity needs significant strategic development to provide safe, affordable, effective, and equitable SOA care to meet its population’s needs. At the national level, first, care capacity at the first level needs to be strengthened. This can improve LCoGS indicators on timeliness, affordability, and surgical volume. SDHs in three to four strategic locations need to be upgraded with the capacity to provide Bellwether procedures. Diabetic foot amputation should be added as a fourth Pacific-specific Bellwether procedure. Strategic locations could include rural areas between the DHs (e.g. Sigatoka and Rakiraki) and high-density urban areas along the Suva-Nausori corridor to offload the national referral hospital. Outreach programs can improve preparedness for bellwether upgrades and emergency deployment in response to natural disasters and pandemics.

Second, capacity constraints in DHs need to be overcome to improve surgical volume and reduce regional inequity. There needs to be sustained growth in a skilled SOA workforce. Although only the SOA specialist density is measured by the LCoGS indicators, previous studies in the Pacific highlighted the importance of growing an inclusive, multidisciplinary nursing and allied health workforce [4,5]. Existing ORs must be upgraded and maintained to function at maximal capacity with improved climate change adaptation and resilience.

Third, the WHO building blocks must be coordinated to improve SOA care across all levels. The procurement and supply chain process needs to be reformed, consistent with existing health strategy. Primary and public health efforts should be strengthened to prevent diabetic-related surgical complications. Overall, substantial increased financial investment in SOA care is required. This should arise both from increased CHE as a percentage of GDP domestically and funding support from donors and partners internationally.

**Panel 1:** Policy recommendations at the national, intergovernmental, and international levels and for the private sector.

National		
Infrastructure	Workforce	Information management
Strengthen facility maintenance and climate resilience Improve consumable and pharmaceutical procurement and supply chain	Growing and maintaining a skilled SOA workforce Inclusive, multidisciplinary workforce: SOA specialists, nurses, allied health. Create more specialist posts to reduce attrition Allow part-time and flexible posts	Prospective POMR collection
Finance	Governance	
Increase investment in health with increased CHE as a percentage of GDP	Integrate with primary health and public health strategies to prevent diabetes-related surgery Incorporate NSOAP into national strategic plans	
Service delivery		
Bellwether upgrade of three to four SDHs in strategic locations within five to ten years Strengthen outreach programs Strengthen specialised services (subspecialty surgery, intensive care, pathology, radiology, transfusion, biomedical engineering)		
Intergovernmental		
Regional collaboration with neighbouring PICs with Fiji as a hub for 1) overseas referral for specialised services, 2) training, 3) professional associations, such as the Pacific Island Surgical Association		
International		
Financial support by donors for surgical system strengthening Coordinate support to priority areas identified at the national level. Assistance in areas of geographic challenges to PICs, including the maintenance of facility, equipment and workforce, and the provision of specialised services Technical assistance in developing climate-resilient surgical facilities,		
Private sector		
Outsource to the private sector to improve surgical volume and reduce waiting times, paying attention to impacts on affordability and equity.		

CHE, current health expenditure; GDP, gross domestic product; NSOAP, national surgical, obstetric, and anaesthesia plan; POMR, perioperative mortality; SDH, subdivisional hospital; SOA, surgical, obstetric, and anaesthesia.

At an intergovernmental level, as the largest South Pacific nation and an upper-middle-income country, Fiji has the unique potential for facilitating regional collaboration. If Fiji can be internally self-sustainable in its primary, secondary, and tertiary SOA care, it can be a regional referral centre for specialised SOA care. Not only can this help Fiji’s Pacific neighbours reduce the distance and cost of overseas referrals, but it can also provide Fiji with additional revenue and help maintain its specialised SOA workforce. In addition to training, Fiji can be a hub for regional professional associations to facilitate continuous professional development.

International partners can assist in areas of geographic challenge to the Pacific, such as the maintenance of facilities, equipment and workforce, and the provision of specialised services through visiting specialists and regional remote support networks. Technical assistance in developing climate-resilience health facilities has been particularly requested. This can help ORs function in extreme temperatures and avoid utility disruption during extreme weather events in remote Eastern islands. International partners must coordinate their resources and support to priorities identified at the national level.

Since the time of the study, the role of the private sector in Fiji has increased. Outsourcing to the private sector has emerged as a solution for improving surgical volume. However, its impact on access, affordability and equity must be examined.

### Strengths and limitations

Our study is the first comprehensive assessment of SOA capacity in Fiji. Unlike previous studies in the Pacific and globally, it is led by local surgeons and policymakers and is closely integrated into the policy process [4,16–18]. Data is interpreted with the local context in mind, leading to improved internal validity, applicability, and generalisability in the real world [21]. The author research equity reflexivity statement is listed in S2 Appendix. Quantitative results were closely triangulated with qualitative findings and stakeholder meeting proceedings.

Several limitations exist. First, we did not include the private sector. According to information provided by the Fiji Clinical Services Network, 12.5 FTE specialists were estimated to work in the private sector at the time of the study, which increases the national SOA specialist density to 8.6 by 21%. Therefore, the total national SOA specialist density and surgical volume were likely underestimated by a small percentage. Second, two SDHs did not participate; however, this likely had a limited impact on our findings. Third, data were collected during the COVID pandemic, and elective surgical volume may have been lower than usual. Lastly, the SAT did not capture all allied health personnel and essential surgical procedures, such as otorhinolaryngology procedures.

Future studies in Fiji should include private facilities, assess climate change adaptation, and monitor care quality with service expansion. Our study design sets an example for future global surgery studies in terms of local contextualisation, integration with policy, and a strength-based approach.

## Conclusions

This study identified significant gaps in infrastructure, workforce, and service delivery with variations by geographic division and care level. Although there has been encouraging growth in SOA specialist density and surgical volume, significant further strategic development of SOA care capacity in Fiji is required to deliver timely, effective, and equitable care suited to the needs of its population and fulfil its potential as a hub for regional collaboration. Policy interventions at the national, intergovernmental, international, and private sector levels need to be epidemiologically, geographically, environmentally, and culturally contextualised. Key recommendations include SDH capacity strengthening, SOA workforce development, facility maintenance and climate adaptation, underpinned by domestic and international financial investment.

## Supporting information

S1 Table The definitions and examples of primary, secondary, and tertiary procedures. (DOCX)

S2 Table Equipment, supplies, and medication availabilities across public health facilities in Fiji, 2021. (DOCX)

S3 Table Breakdown of surgeons by specialty, Fiji, 2021. (DOCX)

S4 Table The ten most commonly performed secondary and tertiary operation, Fiji, 2021. (DOCX)

S1 Appendix World Health Organization – Program in Global Surgery and Social Change (WHO-PGSSC) Surgical Assessment Tool. (DOCX)

S2 Appendix Author reflexivity statement. (DOCX)

## References

[1] World Health Organization. WHA 68.15: strengthening emergency and essential surgical care and anaesthesia as a component of universal health coverage. Geneva, Switzerland: World Health Organization; 2015.

[2] World Health Organization. Outcomes of the Thirteenth Pacific Health Ministers Meeting. Tahiti, French Polynesia: World Health Organization; 2019.

[3] Sarfati D, Dyer R, Sam FA-L, Barton M, Bray F, Buadromo E, et al. Cancer control in the Pacific: big challenges facing small island states. Lancet Oncol. 2019;20(9):e475–92. doi: 10.1016/S1470-2045(19)30400-0 31395476 PMC7746436

[4] Young S, Perry WRG, Leodoro B, Nosa V, Bissett I, Windsor JA, et al. Challenges and opportunities in the provision of surgical care in Vanuatu: a mixed methods analysis. World J Surg. 2016;40(8):1865–73. doi: 10.1007/s00268-016-3535-9 27142621

[5] Qin RX, Zhang G, Lim MX, Waqainabete I, Tudravu J, Turagava J, et al. Assessment of essential surgical and anaesthesia care capacity: a cross-sectional study in five Pacific Island Countries. Lancet Reg Health West Pac. 2023;39:100830. doi: 10.1016/j.lanwpc.2023.100830 37484709 PMC10362349

[6] Qin RX, Fowler ZG, Jayaram A, Stankey M, Yoon S, McLeod E, et al. The current status of surgical care in the Asia-Pacific region and opportunities for improvement: proceedings. BMC Proc. 2023;17(Suppl 5):12. doi: 10.1186/s12919-023-00255-0 37488551 PMC10367230

[7] Guest GD, McLeod E, Perry WRG, Tangi V, Pedro J, Ponifasio P, et al. Collecting data for global surgical indicators: a collaborative approach in the Pacific Region. BMJ Glob Health. 2017;2(4):e000376. doi: 10.1136/bmjgh-2017-000376 29225948 PMC5717952

[8] Fiji Bureau of Statistics. 2017 Population and Housing Census Release 1. Suva, Fiji; 2018.

[9] Britton SG. The evolution of a colonial space-economy: the case of Fiji. J Hist Geogr. 1980;6(3):251–74. doi: 10.1016/0305-7488(80)90081-x

[10] World Bank. Population, total - Fiji | Data. [cited 2021 Oct 16]. Available: https://data.worldbank.org/indicator/SP.POP.TOTL?locations=FJ

[11] Parks T, Kado J, Miller AE, Ward B, Heenan R, Colquhoun SM, et al. Rheumatic heart disease-attributable mortality at ages 5-69 years in Fiji: a five-year, national, population-based record-linkage Cohort study. PLoS Negl Trop Dis. 2015;9(9):e0004033. doi: 10.1371/journal.pntd.0004033 26371755 PMC4570761

[12] Vodonaivalu L, Bullen C. Trends in cervical cancer in Fiji, 2000-2010. Public Health Action. 2013;3(1):68–71. doi: 10.5588/pha.12.0066 26392999 PMC4463084

[13] Bythell M, Fong J, Vuadreu R. Maternal mortality in Fiji 2008-2012. Fiji J Public Health. 2014;3:32–7.

[14] Wainiqolo I, Kafoa B, Kool B, Herman J, McCaig E, Ameratunga S. A profile of injury in Fiji: findings from a population-based injury surveillance system (TRIP-10). BMC Public Health. 2012;12:1074. doi: 10.1186/1471-2458-12-1074 23234597 PMC3540002

[15] Khan S, Mohammadnezhad M, Ratu A, Ghosh A, Ali W, Nand D, et al. Patterns and risk factors associated with index Lower Extremity Amputations (LEA) among Type 2 Diabetes Mellitus (T2DM) patients in Fiji. Prim Care Diabetes. 2021;15(6):1012–8. doi: 10.1016/j.pcd.2021.07.007 34284950

[16] Natuzzi ES, Kushner A, Jagilly R, Pickacha D, Agiomea K, Hou L, et al. Surgical care in the Solomon Islands: a road map for universal surgical care delivery. World J Surg. 2011;35(6):1183–93. doi: 10.1007/s00268-011-1097-4 21487845

[17] Comery B, Perry WRG, Young S, Dare A, Matalavea B, Bissett IP, et al. Delivery of surgical care in Samoa: perspectives on capacity, barriers and opportunities by local providers. ANZ J Surg. 2020;90(10):1910–4. doi: 10.1111/ans.15295 31210420

[18] Martin J, Tau G, Cherian MN, Vergel de Dios J, Mills D, Fitzpatrick J, et al. Survey of the capacity for essential surgery and anaesthesia services in Papua New Guinea. BMJ Open. 2015;5(12):e009841. doi: 10.1136/bmjopen-2015-009841 26674504 PMC4691725

[19] Blair KJ, Paladino L, Shaw PL, Shapiro MB, Nwomeh BC, Swaroop M. Surgical and trauma care in low- and middle-income countries: a review of capacity assessments. J Surg Res. 2017;210:139–51. doi: 10.1016/j.jss.2016.11.005 28457320

[20] Qin R, Alayande B, Okolo I, Khanyola J, Jumbam DT, Koea J, et al. Colonisation and its aftermath: reimagining global surgery. BMJ Glob Health. 2024;9(1):e014173. doi: 10.1136/bmjgh-2023-014173 38176746 PMC10773343

[21] Abimbola S. The uses of knowledge in global health. BMJ Glob Health. 2021;6(4):e005802. doi: 10.1136/bmjgh-2021-005802 33820807 PMC8030475

[22] Roberts G, Wayne I, Tuiketei T, Nadakuitavuki R, Otealagi S, Singh S, et al. The Fiji Islands health system review. Geneva, Switzerland: The World Health Organization; 2011.

[23] Ministry of Health & Medical Services. Strategic Plan 2020–2025. Suva, Fiji; 2020.

[24] Naidu V, Matadradra A, Sahib M, Osborne J. Informal settlements and social inequality in Fiji: evidence of serious policy gaps. Pac Stud. 2015;35:27–42.

[25] Ministry of Health and Medical Services. Fiji National Health Accounts - National Health Expenditure 2016–2021. Suva, Fiji; 2021.

[26] Lin Y, Raykar NP, Saluja S, Mukhopadhyay S, Sharma S, Frett B, et al. Identifying essential components of surgical care delivery through quality improvement: an updated surgical assessment tool. Int J Surg. 2020;82:103–7. doi: 10.1016/j.ijsu.2020.08.002 32810595

[27] Meara JG, Leather AJM, Hagander L, Alkire BC, Alonso N, Ameh EA, et al. Global Surgery 2030: evidence and solutions for achieving health, welfare, and economic development. Lancet. 2015;386(9993):569–624. doi: 10.1016/S0140-6736(15)60160-X 25924834

[28] Debas HT, Donkor P, Gawande A, Jamison DT, Kruk ME, Mock CN, editors. Essential surgery: disease control priorities, third edition (volume 1). Washington (DC): World Bank; 2015. 26740991

[29] Siddiqi S, Khan MS, Rizvi N, Naeem I, Rozi S, Enam A, et al. Are rural hospitals in Pakistan responding to the global surgery movement? An analysis of the gaps, challenges and opportunities. World J Surg. 2020;44(4):1045–52. doi: 10.1007/s00268-019-05327-x 31848676

[30] Betran AP, Torloni MR, Zhang JJ, Gülmezoglu AM, WHO Working Group on Caesarean Section. WHO statement on caesarean section rates. BJOG. 2016;123(5):667–70. doi: 10.1111/1471-0528.13526 26681211 PMC5034743

[31] Tin STW, Lee CMY, Colagiuri R. A profile of diabetes in Pacific Island Countries and Territories. Diabetes Res Clin Pract. 2015;107(2):233–46. doi: 10.1016/j.diabres.2014.10.010 25467624

[32] Kumar K, Snowdon W, Ram S, Khan S, Cornelius M, Tukana I, et al. Descriptive analysis of diabetes-related amputations at the Colonial War Memorial Hospital, Fiji, 2010-2012. Public Health Action. 2014;4(3):155–8. doi: 10.5588/pha.14.0026 26400802 PMC4533803

[33] World Bank. Current health expenditure (%GDP) - Fiji. [cited 5 Mar 2021]. Available from: https://data.worldbank.org/indicator/SH.XPD.CHEX.GD.ZS?locations=NZ-FJ

[34] World Bank. GDP per capita (current US$) - Fiji. In: World Bank [Internet]. [cited 2022 May 13]. Available from: https://data.worldbank.org/indicator/NY.GDP.PCAP.CD?locations=FJ

