# Supplementary material for: A cross-sectional study of essential surgical, obstetric, and anaesthesia care capacity in the public sector in Fiji
Source: PLOS Glob Public Health. 2025 Feb 5;5(2):e0003829. doi: 10.1371/journal.pgph.0003829 (PMC11798476; doi:10.1371/journal.pgph.0003829)
Supplement: S1 Table — (DOCX) [file pgph.0003829.s001.docx]

## S1 Table. The definitions and examples of primary, secondary, and tertiary procedures

| Procedures | Level of hospital care | Definition | Example procedures from the WHO-PGSSC SAT |
| --- | --- | --- | --- |
| Primary | First-level level | Often only one general practice physician or a nonphysician clinician Limited laboratory services available for general analysis but not for specialized pathological analysis | Normal obstetric delivery  Management of non-displaced fractures  Drainage of superficial abscess  Suturing laceration  Wound debridement  Biopsy (lymph node, mass, other) Removal of foreign body (throat/eye/ear/nose)  Male circumcision |
| Secondary | Second-level level | More differentiated by function, with as many as 5 to 10 clinical specialties | Caesarean birth  Vacuum extraction/forceps delivery  Hysterectomy  Tubal ligation  Manual vacuum aspiration and dilation and curettage  Ectopic pregnancy  Inspection with acetic acid, cryotherapy for cervical lesions  General Surgery  Appendectomy  Gallbladder disease  Hernia, including incarceration  Bowel obstruction  Colostomy/ileostomy  Vasectomy  Repair of intestinal perforations  Hydrocelectomy  Relief of urinary obstruction  Injury / Orthopaedic  Fracture reduction  Irrigation and debridement of open fractures  Trauma laparotomy  Placement of external fixator  Tube thoracostomy  Amputations  Escharotomy/fasciotomy  Skin grafting  Burr hole  2Resuscitation with advanced life support measures, including surgical airway  Drainage of septic arthritis  Debridement of osteomyelitis |
| Tertiary | Third-level level | Highly specialized staff and technical equipment—for example, cardiology, intensive care unit, and specialized imaging units Clinical services highly differentiated by function Teaching activities in some facilities | Repair obstetric fistulaRepair of cleft lip and palateRepair of club footShunt for hydrocephalusRepair of anorectal malformation and Hirschsprung’s DiseaseCataract extraction and insertion of intraocular lensEyelid surgery for trachoma |
