# Supplementary material for: A cross-sectional study of essential surgical, obstetric, and anaesthesia care capacity in the public sector in Fiji
Source: PLOS Glob Public Health. 2025 Feb 5;5(2):e0003829. doi: 10.1371/journal.pgph.0003829 (PMC11798476; doi:10.1371/journal.pgph.0003829)
Supplement: S2 Table — (DOCX) [file pgph.0003829.s002.docx]

S2 Table. Equipment, supplies, and medication availabilities across public health facilities in Fiji, 2021

|  | | *Surgical Equipment* | | | *Anaesthetic Equipment* | | | *Supplies* | | | *Medication* | |
| --- | --- | --- | --- | --- | --- | --- | --- | --- | --- | --- | --- | --- |
| *Facility* | Population | Light Source | Scalpel handle | Steriliser | Adult BMV | Paeds BMV | Difficult airway set | Chest Drain | Sterile Gloves | Sutures | Inhalational Anaesthesia | Antibiotics |
| **Divisional Hospitals (DHs)** | | | | | | | | | | | | |
| CWM | 415,932 |  |  |  |  |  |  |  |  |  |  |  |
| Lautoka | 337,041 |  |  |  |  |  |  |  |  |  |  |  |
| Labasa | 131,914 |  |  |  |  |  |  |  |  |  |  |  |
| **All DHs** | | 3/3 | 3/3 | 3/3 | 3/3 | 3/3 | 3/3 | 3/3 | 3/3 | 3/3 | 3/3 | 3/3 |
| **Subdivisional Hospitals (SDHs)** | | | | | | | | | | | | |
| **Central Division** | | | | | | | | | | | | |
| Navua | 27,895 |  |  |  |  |  |  |  |  |  |  |  |
| Korovou | 22,649 |  |  |  |  |  |  |  |  |  |  |  |
| Vunidawa | 17,769 |  |  |  |  |  |  |  |  |  |  |  |
|  |  | 67% | 67% | 67% | 67% | 67% | 33% | 0 | 67% | 33% | 0% | 67% |
| **Eastern Division** | | | | | | | | | | | | |
| Levuka | 15,657 |  |  |  |  |  |  |  |  |  |  |  |
| Vunisea | 10,869 |  |  |  |  |  |  |  |  |  |  |  |
| Lakeba | 4,642 |  |  |  |  |  |  |  |  |  |  |  |
| Lomaloma | 2,781 |  |  |  |  |  |  |  |  |  |  |  |
| Rotuma | 1583 |  |  |  |  |  |  |  |  |  |  |  |
|  |  | 80% | 60% | 100% | 80% | 80% | 20% | 0% | 100% | 80% | 20% | 80% |
| **Western Division** | | | | | | | | | | | | |
| Nadi | 75,838 |  |  |  |  |  |  |  |  |  |  |  |
| Ba | 72,582 |  |  |  |  |  |  |  |  |  |  |  |
| Sigatoka | 58,940 |  |  |  |  |  |  |  |  |  |  |  |
| Rakiraki | 30,416 |  |  |  |  |  |  |  |  |  |  |  |
|  |  | 50% | 25% | 50% | 25% | 25% | 25% | 0% | 25% | 25% | 25% | 100% |
| **Northern Division** | | | | | | | | | | | | |
| Savusavu | 33,660 |  |  |  |  |  |  |  |  |  |  |  |
| Taveuni | 16,787 |  |  |  |  |  |  |  |  |  |  |  |
| Nabouwalu | 15,489 |  |  |  |  |  |  |  |  |  |  |  |
|  |  | 67% | 67% | 100% | 100% | 67% | 0% | 67% | 67% | 67% | 33% | 67% |
| **All SDHs** | | 10/15 | 8/15 | 12/15 | 10/15 | 9/15 | 3/15 | 2/15 | 10/15 | 8/15 | 3/15 | 12/15 |

BMV: bag mask ventilation; CWM: Colonial War Memorial; DH: divisional hospital
