# Supplementary material for: A cross-sectional study of essential surgical, obstetric, and anaesthesia care capacity in the public sector in Fiji
Source: PLOS Glob Public Health. 2025 Feb 5;5(2):e0003829. doi: 10.1371/journal.pgph.0003829 (PMC11798476; doi:10.1371/journal.pgph.0003829)
Supplement: S3 Table — (DOCX) [file pgph.0003829.s003.docx]

S3_Table.

Breakdown of surgeons by specialty, Fiji, 2021

| Specialty | DH #1 CWMH | | DH #2 Lautoka | | DH #3 Labasa |
| --- | --- | --- | --- | --- | --- |
|  | Full-time | Part-time | Full-time | Part-time | Full-time |
| General surgery | 2 | 3 | 2 |  | 2 |
| Orthopaedics | 1 | 1 | 2 |  | 1 |
| Neurosurgery | 1 |  |  |  |  |
| Urology | 1 | 1 |  |  |  |
| Paediatric surgery | 2 |  |  |  |  |
| Ear, nose, throat | 1 |  |  |  |  |
| Plastics | 1 |  |  |  |  |
| Maxillo-facial surgery | 1 | 1 |  |  |  |
| Ophthalmology | 4 |  |  |  | 1 |

CWM: Colonial War Memorial; DH: divisional hospital
