# Supplementary material for: A cross-sectional study of essential surgical, obstetric, and anaesthesia care capacity in the public sector in Fiji
Source: PLOS Glob Public Health. 2025 Feb 5;5(2):e0003829. doi: 10.1371/journal.pgph.0003829 (PMC11798476; doi:10.1371/journal.pgph.0003829)
Supplement: S4 Table — (DOCX) [file pgph.0003829.s004.docx]

S4 Table.

The ten most commonly performed secondary and tertiary operation*, Fiji, 2021

| Rank | Type of Surgical Operation | Number | Percentage |
| --- | --- | --- | --- |
| 1 | Caesarean Section | 3439 | 17.3% |
| 2 | Open fracture management | 996 | 5.0% |
| 3 | Amputations | 950 | 4.8% |
| 4 | Laparotomy | 830 | 4.2% |
| 5 | Fracture reduction | 694 | 3.5% |
| 6 | Paediatric surgery | 634 | 3.2% |
| 7 | Gallbladder surgery | 342 | 1.7% |
| 8 | Appendectomy | 341 | 1.7% |
| 9 | Ectopic pregnancy surgery | 284 | 1.4% |
| 10 | Hernia repair | 269 | 1.4% |

*procedures performed at primary care centres are excluded
