# Supplementary material for: A cross-sectional study of essential surgical, obstetric, and anaesthesia care capacity in the public sector in Fiji
Source: PLOS Glob Public Health. 2025 Feb 5;5(2):e0003829. doi: 10.1371/journal.pgph.0003829 (PMC11798476; doi:10.1371/journal.pgph.0003829)
Supplement: S1 Appendix — (DOCX) [file pgph.0003829.s005.docx]

## S1 Appendix.

## World Health Organization – Program in Global Surgery and Social Change (WHO-PGSSC) Surgical Assessment Tool

| GENERAL QUESTIONS | | |
| --- | --- | --- |
|  | State / Jurisdiction / Municipality: | |
|  | Name of healthcare facility: | |
|  | Address of healthcare facility: | |
|  | Phone number and email of healthcare facility: | |
|  | Date of data collection (dd/mm/yyyy): | |
|  | Name and professional title of staff filling out form:: | |
|  | Contact information of staff completing this assessment (phone and email):: | |
|  | Level of facility being evaluated | Health Centre / Clinic  District/Rural Hospital / First Referral Hospital  Provincial/Secondary Hospital  Tertiary/Teaching/Specialized Hospital  Other: |
|  | Type of facility being evaluated (all that apply) | Public Private NGO Mission Other: |
|  | Is surgical care provided with surgical missions or campaigns? | Yes, all surgical care Yes, some surgical care No |

| INFRASTRUCTURE | | | |  |  |
| --- | --- | --- | --- | --- | --- |
| **General Infrastructure - How often is this item available and functional?** | | | |  |  |
|  | Electricity/operational power generator | 0 (Never) 1-25% (Rarely)  26-50% (Sometimes) 51-75% (Often)  76-99% (Almost always) 100% (Always) | |  |  |
|  | Running water | 0 (Never) 1-25% (Rarely)  26-50% (Sometimes) 51-75% (Often)  76-99% (Almost always) 100% (Always) | |  |  |
|  | Internet | 0 (Never) 1-25% (Rarely)  26-50% (Sometimes) 51-75% (Often)  76-99% (Almost always) 100% (Always) | |  |  |
|  | Oxygen | 0 (Never) 1-25% (Rarely)  26-50% (Sometimes) 51-75% (Often)  76-99% (Almost always) 100% (Always) | |  |  |
|  |  | **Last 365 days** |  |  |  |
|  | Total number of inpatient hospital beds |  |  |  |  |
|  | Total number of surgical beds (if specified) |  |  |  |  |
|  | Total number of post-anaesthesia care beds |  |  |  |  |
|  | Total number of functioning operating rooms (major and minor) |  |  |  |  |
|  | Total number of advanced care/ICU beds |  |  |  |  |
|  | Total number of functional ventilators in the ICU |  |  |  |  |
|  | Total number of admissions in a year (all specialties) |  |  |  |  |
|  | Total number of outpatients seen in a year (all specialties) |  |  |  |  |
| **Pharmacy: How often are the following available for surgery?**   \| Inhalational general anaesthesia \| 0 (Never) 1-25% (Rarely)  26-50% (Sometimes) 51-75% (Often)  76-99% (Almost always) 100% (Always) \| \| --- \| --- \| \| IV sedation anaesthesia (Ketamine, Midazolam, Propofol, etc) \| 0 (Never) 1-25% (Rarely)  26-50% (Sometimes) 51-75% (Often)  76-99% (Almost always) 100% (Always) \| \| Spinal anaesthesia \| 0 (Never) 1-25% (Rarely)  26-50% (Sometimes) 51-75% (Often)  76-99% (Almost always) 100% (Always) \| \| Regional anaesthesia \| 0 (Never) 1-25% (Rarely)  26-50% (Sometimes) 51-75% (Often)  76-99% (Almost always) 100% (Always) \| \| Peri-operative antibiotics \| 0 (Never) 1-25% (Rarely)  26-50% (Sometimes) 51-75% (Often)  76-99% (Almost always) 100% (Always) \| \| IV fluids \| 0 (Never) 1-25% (Rarely)  26-50% (Sometimes) 51-75% (Often)  76-99% (Almost always) 100% (Always) \| \| Muscle relaxants/paralytics \| 0 (Never) 1-25% (Rarely)  26-50% (Sometimes) 51-75% (Often)  76-99% (Almost always) 100% (Always) \| \| Sedatives \| 0 (Never) 1-25% (Rarely)  26-50% (Sometimes) 51-75% (Often)  76-99% (Almost always) 100% (Always) \| \| Vasopressors \| 0 (Never) 1-25% (Rarely)  26-50% (Sometimes) 51-75% (Often)  76-99% (Almost always) 100% (Always) \| \| Post-operative narcotics \| 0 (Never) 1-25% (Rarely)  26-50% (Sometimes) 51-75% (Often)  76-99% (Almost always) 100% (Always) \| \| **Radiology: How often do you have access to functioning radiology equipment?** \| \| | | | |  |  |
|  | X-ray machine | 0 (Never) 1-25% (Rarely)  26-50% (Sometimes) 51-75% (Often)  76-99% (Almost always) 100% (Always) | |  |  |
|  |   Ultrasound | 0 (Never) 1-25% (Rarely)  26-50% (Sometimes) 51-75% (Often)  76-99% (Almost always) 100% (Always) | |  |  |
|  |   CT scanner | 0 (Never) 1-25% (Rarely)  26-50% (Sometimes) 51-75% (Often)  76-99% (Almost always) 100% (Always) | |  |  |
|  |   MRI scanner | 0 (Never) 1-25% (Rarely)  26-50% (Sometimes) 51-75% (Often)  76-99% (Almost always) 100% (Always) | |  |  |
| **Blood Supply** | | | |  |  |
|  | How often are you able to administer a blood transfusion within 2 hours in your facility? | 0 (Never) 1-25% (Rarely)  26-50% (Sometimes) 51-75% (Often)  76-99% (Almost always) 100% (Always) | |  |  |
| **Laboratory** | | | |  |  |
|  | How often is the lab able to run a Complete Blood Count (haemoglobin, haematocrit, WBC, platelets)? | 0 (Never) 1-25% (Rarely)  26-50% (Sometimes) 51-75% (Often)  76-99% (Almost always) 100% (Always) | |  |  |
|  | How often is the lab able to run a chemistry panel (BUN, creatinine, Na, K, etc.)? | 0 (Never) 1-25% (Rarely)  26-50% (Sometimes) 51-75% (Often)  76-99% (Almost always) 100% (Always) | |  |  |
|  | How often is the lab able to run coagulation studies (PT, PTT, BT, INR)? | 0 (Never) 1-25% (Rarely)  26-50% (Sometimes) 51-75% (Often)  76-99% (Almost always) 100% (Always) | |  |  |
|  | How often is the lab able to do a urinalysis? | 0 (Never) 1-25% (Rarely)  26-50% (Sometimes) 51-75% (Often)  76-99% (Almost always) 100% (Always) | |  |  |
|  |   How often are you able to screen for an infectious panel (HIV, hepatitis virus)? | 0 (Never) 1-25% (Rarely)  26-50% (Sometimes) 51-75% (Often)  76-99% (Almost always) 100% (Always) | |  |  |
| **Access and referral systems** | | | |  |  |
|  | What is the population served by this facility? |  | |  | |
|  | What percentage of your patients can reach the hospital within 2 hours of travel? | 0 (Never) 1-25% (Rarely)  26-50% (Sometimes) 51-75% (Often)  76-99% (Almost always) 100% (Always) | |  | |
|  | Total number of patients that you refer for surgical evaluation to a higher level facility per year |  | |  | |

| **SERVICE DELIVERY** | | | |
| --- | --- | --- | --- |
| **Surgical volume** | | | |
|  | How many of the following procedures have been performed at this facility? | | **Last 365 days** |
|  | Total number of surgeries | | # |
|  | Number of caesarean deliveries | | # |
|  | Number of laparotomies | | # |
|  | Number of open fracture repairs | | # |
|  | Number of paediatric surgeries (<15 years) | | # |
|  | Number of emergent / urgent (non-elective) cases | | # |
|  |  | | |
| **Primary Procedures** | | |  |
|  | 1. Normal obstetric delivery | | # |
|  | 2. Management of non-displaced fractures | | # |
|  | 3. Drainage of superficial abscess | | # |
|  | 4. Suturing laceration | | # |
|  | 5**.** Wound debridement | | # |
|  | 6**.** Biopsy (lymph node, mass, other) | | # |
|  | 7. Removal of foreign body (throat/eye/ear/nose) | | # |
|  | 8. Male circumcision | | # |
|  | | | |
|  | | | |
| **District Hospital – Secondary Procedures** | | | |
|  | How many of the following procedures have been performed at this facility? | | |
| **Obstetrics and Gynaecology** | | | |
|  | **1.** Caesarean birth | | # |
|  | 2. Vacuum extraction/forceps delivery | | # |
|  | 3. Hysterectomy | | # |
|  | 4. Tubal ligation | | # |
|  | 5. Manual vacuum aspiration and dilation and curettage | | # |
|  | 6. Ectopic pregnancy | | # |
|  | 8. Inspection with acetic acid, cryotherapy for cervical lesions | |  |
| **General Surgery** | | | |
|  | 10. Appendectomy | | # |
|  | 11. Gallbladder disease | | # |
|  | 14. Hernia, including incarceration | | # |
|  | 13. Bowel obstruction | | # |
|  | 14. Colostomy/ileostomy | | # |
|  | 15. Vasectomy | | # |
|  | 16. Repair of intestinal perforations | | # |
|  | 15. Hydrocelectomy | | # |
|  | 16. Relief of urinary obstruction | | # |
| **Injury / Orthopaedic** | | | |
|  | 17. Fracture reduction | | # |
|  | 18. Irrigation and debridement of open fractures | | # |
|  | 19. Trauma laparotomy | | # |
|  | 20. Placement of external fixator | | # |
|  | 21. Tube thoracostomy | | # |
|  | 22. Amputations | | # |
|  | 23. Escharotomy/fasciotomy | | # |
|  | 24. Skin grafting | | # |
|  | 25. Burr hole | | # |
|  | 26. Resuscitation with advanced life support measures, including surgical airway | | # |
|  | 27. Drainage of septic arthritis | | # |
|  | 28. Debridement of osteomyelitis | | # |
| **Referral and Specialised Hospital – Tertiary Procedures** | | | |
|  | 1. Repair obstetric fistula | | # |
|  | 2. Repair of cleft lip and palate | | # |
|  | 3. Repair of club foot | | # |
|  | 4. Shunt for hydrocephalus | | # |
|  | 5. Repair of anorectal malformation and Hirschsprung’s Disease | | # |
|  | 6. Cataract extraction and insertion of intraocular lens | | # |
|  | 7. Eyelid surgery for trachoma | | # |
| **Quality and Safety** | | | |
|  | What is the average number of peri-operative, in-hospital deaths per year? | | # |
|  | How often is the WHO surgical safety checklist utilized in the operating rooms? | | 0 (Never) 1-25% (Rarely)  26-50% (Sometimes) 51-75% (Often)  76-99% (Almost always) 100% (Always) |
|  | How often is pulse oximetry used in the operating theatre? | | 0 (Never) 1-25% (Rarely)  26-50% (Sometimes) 51-75% (Often)  76-99% (Almost always) 100% (Always) |
| **Operating Room Equipment and Supplies –** **How often are the following equipment available and functional for surgery?** | | | |
|  | Total number of functional anaesthesia machines | | # |
|  | Pulse oximetry | | # |
|  | Adult oropharyngeal airway | 0 (Never) 1-25% (Rarely) 26-50% (Sometimes) 51-75% (Often)  76-99% (Almost always) 100% (Always) | |
|  | Paediatric oropharyngeal airway | 0 (Never) 1-25% (Rarely) 26-50% (Sometimes) 51-75% (Often)  76-99% (Almost always) 100% (Always) | |
|  | Adult endotracheal tube | 0 (Never) 1-25% (Rarely) 26-50% (Sometimes) 51-75% (Often)  76-99% (Almost always) 100% (Always) | |
|  | Paediatric endotracheal tube | 0 (Never) 1-25% (Rarely) 26-50% (Sometimes) 51-75% (Often)  76-99% (Almost always) 100% (Always) | |
|  | Adult laryngoscope | 0 (Never) 1-25% (Rarely) 26-50% (Sometimes) 51-75% (Often)  76-99% (Almost always) 100% (Always) | |
|  | Paediatric laryngoscope | 0 (Never) 1-25% (Rarely) 26-50% (Sometimes) 51-75% (Often)  76-99% (Almost always) 100% (Always) | |
|  | Adult facemask bag valve | 0 (Never) 1-25% (Rarely) 26-50% (Sometimes) 51-75% (Often)  76-99% (Almost always) 100% (Always) | |
|  | Paediatric facemask bag valve | 0 (Never) 1-25% (Rarely) 26-50% (Sometimes) 51-75% (Often)  76-99% (Almost always) 100% (Always) | |
|  | Difficult airway kit (LMA) | 0 (Never) 1-25% (Rarely) 26-50% (Sometimes) 51-75% (Often)  76-99% (Almost always) 100% (Always) | |
|  | Adult Magill forceps | 0 (Never) 1-25% (Rarely) 26-50% (Sometimes) 51-75% (Often)  76-99% (Almost always) 100% (Always) | |
|  | Paediatric Magill forceps | 0 (Never) 1-25% (Rarely) 26-50% (Sometimes) 51-75% (Often)  76-99% (Almost always) 100% (Always) | |
|  | Blood pressure monitor or cuff | 0 (Never) 1-25% (Rarely) 26-50% (Sometimes) 51-75% (Often)  76-99% (Almost always) 100% (Always) | |
|  | Pulse oximeter | 0 (Never) 1-25% (Rarely) 26-50% (Sometimes) 51-75% (Often)  76-99% (Almost always) 100% (Always) | |
|  | Stethoscope | 0 (Never) 1-25% (Rarely) 26-50% (Sometimes) 51-75% (Often)  76-99% (Almost always) 100% (Always) | |
|  | Suction apparatus | 0 (Never) 1-25% (Rarely) 26-50% (Sometimes) 51-75% (Often)  76-99% (Almost always) 100% (Always) | |
|  | Thermometer | 0 (Never) 1-25% (Rarely) 26-50% (Sometimes) 51-75% (Often)  76-99% (Almost always) 100% (Always) | |
|  | Nasogastric Tube | 0 (Never) 1-25% (Rarely) 26-50% (Sometimes) 51-75% (Often)  76-99% (Almost always) 100% (Always) | |
|  | Light source | 0 (Never) 1-25% (Rarely) 26-50% (Sometimes) 51-75% (Often)  76-99% (Almost always) 100% (Always) | |
|  | Chest tube | 0 (Never) 1-25% (Rarely) 26-50% (Sometimes) 51-75% (Often)  76-99% (Almost always) 100% (Always) | |
|  | Electrocautery | 0 (Never) 1-25% (Rarely) 26-50% (Sometimes) 51-75% (Often)  76-99% (Almost always) 100% (Always) | |
|  | Autoclave / Sterilizer | 0 (Never) 1-25% (Rarely) 26-50% (Sometimes) 51-75% (Often)  76-99% (Almost always) 100% (Always) | |
|  | Forceps | 0 (Never) 1-25% (Rarely) 26-50% (Sometimes) 51-75% (Often)  76-99% (Almost always) 100% (Always) | |
|  | Syringes with needles | 0 (Never) 1-25% (Rarely) 26-50% (Sometimes) 51-75% (Often)  76-99% (Almost always) 100% (Always) | |
|  | Scalpel | 0 (Never) 1-25% (Rarely) 26-50% (Sometimes) 51-75% (Often)  76-99% (Almost always) 100% (Always) | |
|  | Scissors | 0 (Never) 1-25% (Rarely) 26-50% (Sometimes) 51-75% (Often)  76-99% (Almost always) 100% (Always) | |
|  | Needle holder | 0 (Never) 1-25% (Rarely) 26-50% (Sometimes) 51-75% (Often)  76-99% (Almost always) 100% (Always) | |
|  | Retractor | 0 (Never) 1-25% (Rarely) 26-50% (Sometimes) 51-75% (Often)  76-99% (Almost always) 100% (Always) | |
|  | Sterile gloves | 0 (Never) 1-25% (Rarely) 26-50% (Sometimes) 51-75% (Often)  76-99% (Almost always) 100% (Always) | |
|  | Urinary catheters | 0 (Never) 1-25% (Rarely) 26-50% (Sometimes) 51-75% (Often)  76-99% (Almost always) 100% (Always) | |
|  | Tourniquet | 0 (Never) 1-25% (Rarely) 26-50% (Sometimes) 51-75% (Often)  76-99% (Almost always) 100% (Always) | |
|  | Face masks | 0 (Never) 1-25% (Rarely) 26-50% (Sometimes) 51-75% (Often)  76-99% (Almost always) 100% (Always) | |
|  | Gowns | 0 (Never) 1-25% (Rarely) 26-50% (Sometimes) 51-75% (Often)  76-99% (Almost always) 100% (Always) | |
|  | Disinfectant hand wash | 0 (Never) 1-25% (Rarely) 26-50% (Sometimes) 51-75% (Often)  76-99% (Almost always) 100% (Always) | |
|  | Sterilizing skin prep | 0 (Never) 1-25% (Rarely) 26-50% (Sometimes) 51-75% (Often)  76-99% (Almost always) 100% (Always) | |
|  | Eye protection | 0 (Never) 1-25% (Rarely) 26-50% (Sometimes) 51-75% (Often)  76-99% (Almost always) 100% (Always) | |
|  | Sharps disposal container | 0 (Never) 1-25% (Rarely) 26-50% (Sometimes) 51-75% (Often)  76-99% (Almost always) 100% (Always) | |
|  | Non-sterile Examination Gloves | 0 (Never) 1-25% (Rarely) 26-50% (Sometimes) 51-75% (Often)  76-99% (Almost always) 100% (Always) | |
|  | Sutures | 0 (Never) 1-25% (Rarely) 26-50% (Sometimes) 51-75% (Often)  76-99% (Almost always) 100% (Always) | |

| **WORKFORCE** | | | | |
| --- | --- | --- | --- | --- |
| **Providers** | | | ****Full-time | Part-time |
|  | What is the total number of surgical specialists? (e.g. neurosurgeon, urologist, ophthalmologist, vascular surgeon, otolaryngologist, plastic surgeon, surgical oncologist, cardiothoracic surgeon, general surgeon, obstetrician-gynaecologist, orthopaedic surgeon, anaesthesiologist, paediatric surgeon, etc) | | # | # |
|  | Number of general surgeons | | # | # |
|  | Number of paediatric surgeons | | # | # |
|  | Number of obstetrician-gynaecologists | | # | # |
|  | Number of anaesthesiologists | | # | # |
|  | Number of orthopaedic surgeons | | # | # |
|  |  | | # | # |
|  | Number of general doctors providing surgery | | # | # |
|  | Number of general doctors providing C-sections (specify if same doctor as above) | | # | # |
|  | Number of general doctors providing anaesthesia | | # | # |
|  | Number of non-physicians providing surgery | | # | # |
|  | Number of non-physicians providing C-sections (specify if same doctor as above) | | # | # |
|  | Number of non-physician providing anaesthesia | | # | # |
|  | Number of midwives | | # | # |
|  | Number of nurses on the surgical wards | | # | # |
|  | Number of physician radiologists | | # | # |
|  | Number of certified pathologists | | # | # |
|  | Number of certified pharmacists | | # | # |
|  | Number of certified biomedical technicians | | # | # |
|  | How often is a surgical provider available 24 hours a day? | 0 (Never) 1-25% (Rarely)  26-50% (Sometimes) 51-75% (Often)  76-99% (Almost always) 100% (Always) | | |
|  | How often is an obstetrics/gynaecology provider available 24 hours a day? | 0 (Never) 1-25% (Rarely)  26-50% (Sometimes) 51-75% (Often)  76-99% (Almost always) 100% (Always) | | |
|  | How often is an anaesthesia provider available 24 hours a day? | 0 (Never) 1-25% (Rarely)  26-50% (Sometimes) 51-75% (Often)  76-99% (Almost always) 100% (Always) | | |
| **Continuing medical education** | | | | |
|  | How often do you offer continuing medical education to your staff each year? | Never Daily/Weekly Monthly Quarterly Yearly | | |

| **INFORMATION MANAGEMENT** | | |
| --- | --- | --- |
| **Information systems** | | |
|  | What is the method of record keeping in your hospital? | Electronic Paper Both None |
|  | Are there personnel in charge of maintaining medical records? | Yes No |
|  | Are charts accessible across multiple visits for the same patient? | Yes No |
|  | How often is data prospectively collected for patient outcomes, such as surgical site infection, post op stroke, DVT, etc.? | 0 (Never) 1-25% (Rarely)  26-50% (Sometimes) 51-75% (Often)  76-99% (Almost always) 100% (Always) |
|  | How often is data prospectively collected for post-operative mortality rate? | 0 (Never) 1-25% (Rarely)  26-50% (Sometimes) 51-75% (Often)  76-99% (Almost always) 100% (Always) |
|  | How often are you required to report information to the Ministry of Health or an equivalent agency? | Never Daily/Weekly Monthly Quarterly Yearly |
|  | Do you use telemedicine? | Yes No |
| **Research agenda** | | |
|  | How often does the hospital participate in quality improvement projects, such as mortality & morbidity conferences? | Never Daily/Weekly Monthly Quarterly Yearly |
|  | How many ongoing research projects does the hospital have? | # |
|  | How many ongoing research projects does the department of surgery have? | # |

| **FINANCING** | | |
| --- | --- | --- |
| **Health financing and accounting** | | |
|  | What percentage of your patients have health insurance? | 0 (Never) 1-25% (Rarely) 26-50% (Sometimes) 51-75% (Often) 76-99% (Almost always) 100% (Always) |
| **Budget allocation** | | |
|  | What is your total annual hospital operating budget? | # |
|  | How much of your annual hospital operating budget is allotted to surgery and anaesthesia? | 0% 1-25% 26-50% 51-75% 76-99% 100% |
| **Cost** | | |
|  | What is the average out-of-pocket cost to a patient for a C section? | # |
|  | Average out-of-pocket cost to a patient for an open fracture repair? | # |
|  | Average out-of-pocket cost to a patient for a laparotomy? | # |
|  | Average out-of-pocket cost to a patient for a CBC? | # |
|  | Average out-of-pocket cost to a patient for a Chest X-ray? | # |
|  | Average out-of-pocket cost to a patient for surgery-associated lodging per day? | # |
|  | Average out-of-pocket cost for patient and family transportation per surgery/hospital stay? | # |
|  | Average out-of-pocket cost to a patient for surgery-associated medication per surgery/hospital stay? | # |
|  | Average out-of-pocket cost to a patient for other necessities (e.g. laundry/food) per surgery/hospital stay? | # |
