# Supplementary material for: A cross-sectional study of essential surgical, obstetric, and anaesthesia care capacity in the public sector in Fiji
Source: PLOS Glob Public Health. 2025 Feb 5;5(2):e0003829. doi: 10.1371/journal.pgph.0003829 (PMC11798476; doi:10.1371/journal.pgph.0003829)
Supplement: S2 Appendix — (DOCX) [file pgph.0003829.s006.docx]

S2. Appendix

Author reflexivity statement

1. How does this study address local research and policy priorities?

This study assessed the surgical, obstetric, and anaesthesia care capacity in Fiji to inform the development of a National Surgical, Obstetric, and Anaesthesia Plan (NSOAP).

1. How were local researchers involved in study design?

The NSOAP policy process was led by IW, the Minister of Health, JT, the Chief Medical Advisor, JT, the chief surgeon, and RP, urologist at CWM Hospital. Research input was provided by RQ and KM. IW, JT, JT, RP, KW, and RQ co-led the study design process.

1. How has funding been used to support the local research team(s)?

This project was unfunded.

1. How are research staff who conducted data collection acknowledged?

Medical officers in each hospital conducted the data collection. They were acknowledged in the acknowledgement section.

1. How have members of the research partnership been provided with access to study data?

All study data were made available to every member of the research staff through a shared Google Drive. At the stakeholder meeting, a summary of the data was made available to more than 100 stakeholders across geographic divisions and hospital levels.

1. How were data used to develop analytical skills within the partnership?

Once the data were collected, two Fijian surgical registrars, AS and JM, were recruited into the team to co-lead data analysis under the supervision of RQ and RP. Their research capacity in data analysis skills was developed over the course of a year.

1. How have research partners collaborated in interpreting study data?

AS and JM curated the data and created figures and tables for interpretation. Regular Zoom meetings were held with IW, JT, JT, and RP, who contributed their insight from a policy perspective to data interpretation. As part of the NSOAP process, a summary of data was presented at the stakeholder meeting, and interpretation was sought from stakeholders.

1. How were research partners supported to develop writing skills?

AS and JM were supported by RQ in developing the first draft of the paper. They received editorial feedback and worked on subsequent drafts.

1. How will research products be shared to address local needs?

The data have already been shared with stakeholders as a part of the NSOAP consultation process. The final research product will be shared with the Clinical Services Network and the Ministry of Health to inform the development of an NSOAP and other policies that improve surgical, obstetric, and anaesthesia care.

1. How is the leadership, contribution and ownership of this work by LMIC researchers recognised within the authorship?

AS and JM are joint first authors. RP is a joint senior author. The project had ownership by the Ministry of Health. IW, JT, and JT were all affiliated with the Ministry of Health and are co-authors of this paper.

1. How have early career researchers across the partnership been included within the authorship team?

The co-first authors, AS and JM, are surgical residents with early exposure to research and health policy.

1. How has gender balance been addressed within the authorship?

RQ, a joint senior author, is female and played a key role in supporting AS and JM’s research capacity development.

1. How has the project contributed to training of LMIC researchers?

The project has contributed to the research capacity development of AS and JM as clinicians with interests in research who can integrate health systems research, health policy, and clinical leadership in their future careers. Our collective journey of working on this project has contributed to health system research and data interpretation skills of IW, JT, JT, and RP.

1. How has the project contributed to improvements in local infrastructure?

We hope to improve local infrastructure in the long term by calling for greater ownership and agency of LMIC governments to contribute funding to strengthening the surgical system.

1. What safeguarding procedures were used to protect local study participants and researchers?

Preliminary analysis results were shared with stakeholders during the policy process. We did not have a strict timeline for writing the academic publication and worked around AS and JM’s work and study schedule to avoid placing an undue burden on local researchers' time.
